# *In vitro *and *in vivo *anti-malarial activity of *Boerhavia elegans *and *Solanum surattense*

**DOI:** 10.1186/1475-2875-9-124

**Published:** 2010-05-12

**Authors:** Ali Ramazani, Sedigheh Zakeri, Soroush Sardari, Nastaran Khodakarim, Navid Dinparas Djadidt

**Affiliations:** 1 Malaria and Vector Research Group (MVRG), Biotechnology Research Center, Institut Pasteur of Iran, Tehran, Iran, Pasteur Avenue, P.O. BOX 1316943551, Tehran, Iran; 2 Drug Discovery and Bioinformatics Group, Biotechnology Research Center of Institut Pasteur of Iran, Tehran, Iran

## Abstract

**Background:**

There is an urgent need to identify new anti-malarial drug targets for both prophylaxis and chemotherapy, due to the increasing problem of drug resistance to malaria parasites. In the present study, the aim was to discover novel, effective plant-based extracts for the activity against malaria.

**Methods:**

Ten plants found in Iran were selected by ethnobotanical survey of medicinal plants. The crude ethanolic extracts were tested for *in vitro *anti-plasmodial activity against two strains of *Plasmodium falciparum*: K1 (chloroquine-resistant strain) and CY27 (chloroquine-sensitive strain), using the parasite lactate dehydrogenase (pLDH) assay. The anti-plasmodial activity of the extracts was also assessed in the 4-day suppressive anti-malarial assay in mice inoculated with *Plasmodium berghei *(ANKA strain). Crude ethanolic extracts showed good anti-plasmodial activity were further fractionated by partitioning in water and dichloromethane.

**Results:**

Of 10 plant species assayed, three species: *Boerhavia elegans *(Choisy), *Solanum surattense *(Burm.f.) and *Prosopis juliflora *(Sw.) showed promising anti-plasmodial activity *in vitro *(IC_50 _≤ 50 μg/ml) and *in vivo *with no toxicity. The dichloromethane fraction of three extracts revealed stronger anti-plasmodial activity than the total extracts.

**Conclusion:**

Anti-plasmodial activities of extracts of *B. elegans *and *S. surattense *are reported for the first time.

## Background

Malaria is one of the oldest recorded diseases in the world. Each year 300 to 500 million new cases are diagnosed and approximately 1.5 million people die of the disease; the majority of them are children [1]. The re-emerging of malaria in many parts of the world is due to the rapid increase of resistance to most of the available anti-malarial drugs, as well as resistance of vectors to insecticides [2,3]. Drug resistant strains of *P. falciparum *have been found in many endemic areas of the world and many of conventional anti-malarial drugs have been associated with treatment failure. Furthermore, the difficulty of creating efficient vaccines and also adverse side-effects of the existing anti-malarial drugs highlight the urgent need for novel, well-tolerated anti-malarial drugs [2] for both prophylaxis and treatment of malaria.

History reveals that plants have always been considered as an important source of medicine against malaria: both quinine and artemisinin have been derived from traditional medicine and plant extracts. Artemisinin derivatives are now recommended by the World Health Organization worldwide [4,5], in combination with other drugs, such as lumefantrine, amodiaquine, mefloquine, sulphadoxine-pyrimethamine (SP), as the first-line treatment of malaria. This fact has encouraged the continuing search for new natural product-derived anti-malarial drugs. In malaria-endemic countries, several plants are utilized in traditional medicine for the treatment of malaria and/or fever. Furthermore, several studies have been undertaken to evaluate not only the inhibitory effects of various plant extracts on *P. falciparum *[6,7] using *in vitro *culture, but also *in vivo *anti-malarial properties on *Plasmodium berghei*-infected mice [8,9].

Malaria is endemic in Iran with 16,000 cases in 2008 (Center for Diseases Management and Control, Ministry of Health and Medical Education, unpublished). Despite intensive efforts to control malaria, the disease continues to be one of the greatest health problems in the south-eastern part of the country. In Iran, *P. falciparum *resistance to CQ has been reported since 1983 [10]. As the intensity of CQ resistance increased, the country implemented a change of first-line anti-malarial treatment in 2007 to a combination of SP-artesunate with artemether-lumefantrine (Coartem^®^), as the second-line drug [11]. Considering the great potential of Iranian plant biodiversity and based on ancient Iranian traditional physician's books [12-15], 10 different species of Iranian medicinal plants were selected in this investigation. The aim of this study was to discover novel, effective plant-based medicines for the treatment of malaria. In light of this, *in vitro *screening the anti-plasmodial properties of these plant species and their possible cytotoxic activities were also determined using the brine shrimp assay [16]. Subsequently, based on *in vitro *results, the active extracts were followed-up in a murine model of malaria.

## Methods

### Selection and collection of plant materials

**Figure 1:**
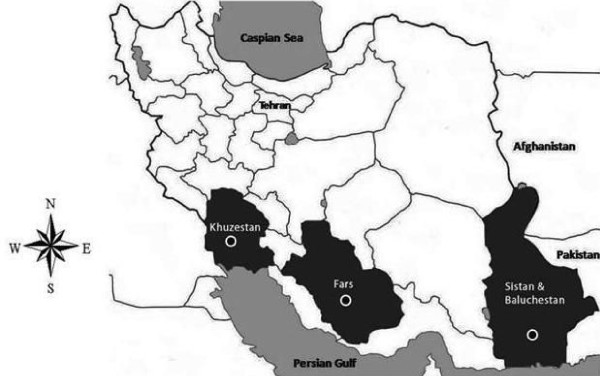
**Map of Iran showing plant collecting places: Sistan and Baluchistan, Khouzestan and Fars Provinces**.

Based on literature and interviews with traditional health practitioners, plants representing five families, seven genera, and 10 species that used against fever, inflammation and microbial infection have been selected. The plants were collected from different parts of Iran, Sistan and Baluchistan, Khouzestan and Fars provinces, from March 2005 to October 2007 (Figure 1). Collections were undertaken from areas endemic for malaria at the present (Sistan and Baluchistan) and that had been endemic in the past (Khouzestan and Fars).

The specimens were identified by Dr. S. Sardari and voucher specimens were deposited at Drug Discovery Group of Biotechnology Research Center at Institut Pasteur of Iran (Table 1). After identification, aerial parts of each specimen were air-dried in shadow at room temperature. The plants were crushed into fine powder using an electric grinder. The powdered samples were stored in appropriate containers and were kept at a cold room (4°C). The studied plant species were indicated in Table 1.

**Table 1:** Names and origins of the selected plants of Iran

Family	Species	Locality (province)	Date of collection	Some traditional uses (Jorjani, 1992; Khorasani, 1992; Avicenna, 2004)	Voucher no.
**Nyctaginaceae**	***Boerhavia elegans***	**S & B**	**March, 2005**	**Dysmenorrhea, urinary tract and intestinal infections, inflammation**	**CH-3**
**Solanaceae**	***Solanum surattense***	**S & B**	**March, 2005**	**Antipyretic, inflammation**	**CH-45**
Solanaceae	*Solanum alatum*	Khouzestan	October, 2007	Headache, infection, wound healing, dysentery	74-10
**Fabaceae**	***Prosopis juliflora***	**Khouzestan**	**October, 2007**	**Fever, inflammation**	**74-32**
Moraceae	*Ficus bengalensis*	Khouzestan	May, 2007	Wound healing, sedative, anti-rheumatism, inflammation	74-31
Moraceae	*Ficus carica*	Khouzestan	May, 2007	Ascites, a Anemia, inflamation	74-46
Rutaceae	*Citrus limon*	Khouzestan	May, 2007	disinfectant, rheumatism, inflammation	74-35
Moraceae	*Morus alba*	Khouzestan	May, 2007	Antipyretic, diuretic, laxative	74-38
Fabaceae	*Acacia farnesiana*	Khouzestan	May, 2007	wound healing, analgesic	74-37
Rutaceae	*Citrus aurantifolia *Swingle	Fars	September, 2007	Disinfectant, sedative, cold, appetite,	78-3

S & B = Sistan and Baluchistan

### Extraction and fractionation

For each plant, 100 g of powdered materials was extracted by percolation three times using 80% ethanol at room temperature. The ethanol extracts were filtered, pooled and dried at 40°C or below using a rotary evaporator. All the extracts were kept in airtight containers and were stored at 4°C for use in anti-plasmodial bioassay and general toxicity tests. Crude ethanolic extracts that showed good anti-plasmodial activity against *P. falciparum *strains, were further fractionated by partitioning in water and dichloromethane. The organic and aqueous phases were concentrated and dried by rotary evaporator and were dissolved in dimethylsulphoxide (DMSO) and double distilled water, respectively, and then stored at -20°C for use in anti-plasmodial assay.

### *Plasmodium falciparum *strains and *in vitro *culture

Laboratory-adapted *P. falciparum *K1 (chloroquine-resistant) and CY27 (chloroquine-sensitive) strains, both originally obtained from Thai patients, were continuously cultured based on a modified method previously described [17]. Briefly, parasites were maintained in continuous culture on human erythrocytes (blood group O^+ ^obtained from the Blood Transfusion Organization, Tehran, Iran), in RPMI 1640 medium supplemented with 10% human AB^+ ^serum, 25 mM N-2-hydroxyethylpiperazine-N-2-ethanesulfonic acid (HEPES), 25 mM NaHCO_3_, and 60 μg/ml gentamicin sulfate, at pH 7.2. The cultures were incubated at 37°C in an atmosphere of 91% N_2_, 6% CO_2 _and 3% O_2_. Parasite cultures were synchronized to the ring stage by treatment with 5% D-sorbitol [18].

### *In vitro *anti-plasmodial assay

Plant extracts were assessed for anti-plasmodial activity *in vitro *using modified parasite lactate dehdrogenase (pLDH) method as described previously [19,20]. Crude plant extracts were first dissolved in DMSO at concentration of 50 mg/ml, sonicated for 10 min and then diluted in malaria culture medium to prepare a 2 mg/ml solution. The highest concentration of solvent that the parasites were exposed to was < 1%, which was shown to have no measurable effect on parasite viability. Microtitration techniques were used to measure the activity of samples over a wide range of concentrations (ranging from 200-1.56 μg/ml). Chloroquine diphosphate and artemisinin (both from Sigma Chemical, USA) were dissolved in double distilled water (1 mg/ml) and DMSO (1 mg/ml), respectively and served as controls in all experiments. All tests were performed in triplicate. Synchronous cultures with parasitaemia of 1% and a final haematocrit of 1.5% were aliquoted into the plates and incubated at 37°C for 72 h. After incubation period, the plates were frozen at -20°C overnight, followed by thawing at room temperature to haemolyze the red blood cells. Parasite growth was determined spectrophotometrically at 650 nm, by measuring the activity of the pLDH in control and drug-treated cultures, using a microplate reader (PowerWave 340, BioTek, USA). At the end of incubation, the cultures were resuspended, and aliquots of 20 μl were removed and added to 100 μl of the Malstat reagent [19,20] in a 96-well microtiter plate. The spectrophotometric assessment of pLDH activity was obtained by adding 25 μl of a solution of 1.9 μM NBT (Nitro Blue Tetrazolium) and 0.24 μM PES (Phenazine Ethosulphate) to the Malstat reagent. The anti-malarial activity of the test compound was expressed as IC_50 _(mean ± S.D. of the least three separate experiments performed in triplicate). The OD values from control wells devoid of plant extracts or drug were referred to as having 100% pLDH activity. The inhibition of each extract or drug concentration was calculated as compared to the untreated control to obtain the IC_50 _values. These values were then expressed as a percentage of 100% growth value and plotted against corresponding concentrations of the drug, using Gene5 micrroplate data collection and analysis software (BioTek, USA) to generate log dose-response curves.

### Brine shrimp toxicity assay

Inhibition of the growth of brine shrimps (*Artemia salina*) was used as a measure of the toxicity of the extracts. The general toxicity in brine shrimp lethality test was carried out according to the methodology described before [16] by using brine shrimp eggs, obtained from Artemia and Aquatic Animals Research Center (Urmia University, Urmia, Iran). The eggs were hatched in artificial sea water (38 g/lit saline) in a vessel described previously [21]. After a 28-30 h hatching period at room temperature, the nauplii were ready for the experiment.

Extracts were prepared by dissolving 50 mg/ml in DMSO and then serially diluted in artificial sea water to the desired concentrations. The final DMSO concentration did not exceed 1%, which was shown not to have any harmful effects on the larvae. Finally, ten shrimps in 100 μl of artificial sea water were added to each vial to give a final volume of 2.5 ml. After 24 h, the survivors were counted under microscope and recorded. Gallic acid (LC_50 _= 20 μg/ml) was used as a positive control, and vials containing shrimps, but without any extracts were included in each test. The toxicity of each extract was determined from the 50% lethality dose (LC_50_) by Finney's Probit analysis [22] of the data created with SPSS 15.0 for windows (SPSS Inc., USA). The general toxicity activity was considered weak when the LC_50 _was between 500 and 1000 μg/ml, moderate when the LC_50 _was between 100 and 500 μg/ml, and strong when the LC_50 _ranged from ≤ 100 μg/ml [23].

### *In vivo *anti-plasmodial assay

The anti-plasmodial activity of the *Prosopis juliflora *(Sw.), *Boerhavia elegans *(Choisy), and *Solanum surattense *(Burm.f.) extracts were assessed by the classical 4-day suppressive test [24,25]. Female BALB/C mice, weight 18-20 g were infected by interaperitoneal (i.p.) inoculation of 10^7 ^infected erythrocytes with CQ-sensitive *P. berghei *(ANKA strain) in a saline suspension of 0.2 ml on the first day (D0) of the experiment.

Plant extracts were solubilized in 18% DMSO or in PBS (water-soluble extract) and administrated within 3 h post-inoculation of mice with the parasite (D0) at different concentrations in a dose volume of 0.2 ml. Groups of five mice were dosed daily by i.p. injection for 4 consecutive days. On day 4, tail blood smear were taken, stained with 10% Giemsa in phosphate buffer, pH 7.2 for 15 min and examined under microscope at 100 ×. The percentage parasitaemia was determined by counting the parasitized red blood cells on at least 3,000 red blood cells. The % suppression of parasitaemia was calculated for each extract by comparing the parasitaemia present in infected controls with those of test mice. Chloroquine diphosphate (25 mg/kg), PBS and DMSO (18%) were used as positive and negative controls, respectively. For all the groups survival time in days was recorded and the mean for each group calculated. The results were analysed statistically using one-way ANOVA and two-tailed Student's *t*-test (SPSS 15.0 Inc., USA) to identify the differences between treated group and controls. The data were considered significant at *P *< 0.05.

## Results

### *In vitro *anti-plasmodial assay

*In vitro *activity of 10 plant extracts against CQ-sensitive (CY27) and -resistant (K1) *P. falciparum *strains are summarized in Table 2. Of 10 extracts tested, three showed significant anti-plasmodial activity at a concentration of 4.68 to 50 μg/ml. Three hydro-alcoholic extracts of *Pr. juliflora*, *B. elegans *and *S. surattense*. showed significant anti-plasmodial activity against both chloroquine-resistant (K1) (IC_50_: 14.78, 15.33 and 50 μg/ml, respectively) and chloroquine-sensitive (CY27) (IC_50_: 4.68, 11.97 and 40.88 μg/ml, respectively). The rest of the other plant extracts showed weak or no activity (IC_50 _> 200 μg/ml) against the studied isolates. The selectivity index (SI) is defined as the ratio of the brine shrimp toxicity to the anti-plasmodial activity and is determined by dividing the LC_50 _values for the brine shrimp nauplii by the IC_50 _value for *P. falciparum*. The extract with higher selectivity (high SI value) indicate potentially safer therapy.

**Table 2:** *In vitro *bioassays on *P. falciparum *CY27 and K1 strains and toxicity assay of the selected plants

	Scientific name		*Artemia salina *assay, LC_50 _(μg/ml)	Anti-plasmodial activity, IC_50 _(μg/ml) ± SD		SI	
		
**No**.	Family	Species		K1	CY27	K1	CY27
**1**	**Nyctaginaceae**	***Boerhavia elegans***	**1020**	**15.33 ± 0.07**	**11.97 ± 0.05**	**18.19**	**23.3**

**2**	**Solanaceae**	***Solanum surattense***	**2507**	**50 ± 0.09**	**40.88 ± 0.84**	**61.32**	**48.86**

3	Solanaceae	*Solanum alatum*	1050	>200	>200	NA	NA

**4**	**Fabaceae**	***Prosopis juliflora***	**1636.68**	**14.78 ± 0.08**	**4.68 ± 0.03**	**110.73**	**349.71**

5	Moraceae	*Ficus bengalensis*	1050	>200	>200	NA	NA

6	Moraceae	*Ficus carica*	130.16	>200	>200	NA	NA

7	Rutaceae	*Citrus limon*	471.39	>200	>200	NA	NA

8	Moraceae	*Morus alba*	256.42	>200	>200	NA	NA

9	Fabaceae	*Acacia farnesiana*	460.12	>200	>200	NA	NA

10	Rutaceae	*Citrus aurantifolia *Swingle	270.17	>200	>200	NA	NA

	Gallic acid		20	ND	ND	NA	NA
	
Controls	Chloroquine		ND	1.25 ± 0.08	0.4 ± 0.01	NA	NA
	
	Artemisinin		ND	0.002 ± 0008	0.007 ± 0.0005	NA	NA

The crude extract of three active extracts (*Pr. juliflora*, *B. elegans *and *S. surattense*) were further fractionated in water and dichloromethane mixture solution and the anti-plasmodial activity of organic and aqueous phases was evaluated against CQ-resistant and -sensitive *P. falciparum *strains. The dichloromethane fraction of the three extracts also showed stronger anti-plasmodial activity than the total extracts (Table 3).

**Table 3:** Anti-plasmodial activity (IC_50_) of water-soluble and dichloromethane fractions of three active plants against *P. falciparum *strains

Plant species	IC_50 _(μg/ml) ± *SD			
	K1		CY27	
	Aqueous	Dichloromethane	Aqueous	Dichloromethane
*B. elegans*	>200	15 ± 0.38	>200	9.37 ± 0.01
*S. surattense*	>200	40 ± 0.16	>200	18.75 ± 0.4
*Pr. juliflora*	32.43 ± 0.2	9.95 ± 0.06	18 ± 0.16	1.4 ± 0.02

*SD = Standard deviation

### Brine shrimp toxicity test

All the ethanolic active extracts were screened against brine shrimp nauplii and the results showed no significant or toxicity activity (LC_50 _> 1,000 μg/ml) for *Pr. juliflora*, *B. elegans*, *S. surattense*, *Solanum alatum *and *Ficus bengalensis*. However, the rest of the extracts showed mild toxicity (LC_50 _100-500 μg/ml) (Table 2).

### *In vivo *anti-malarial activity of *Prosopis juliflora*. *Boerhavia elegans *and *Solanum surattense *on *Plasmodium berghei*

Table 4 shows a summary of parasitaemia suppression (%) for mice on day 4. Three extracts from three plant species showed a significant suppression of parasitaemia (P < 0.05) ranging from 44.1 to 66.2%. In contrast, results with the water-soluble extracts of *Pr. juliflora *were non-significant (P > 0.05) (Table 4). Based on day 10 post-infection, *S. surattense *extract gave 80%, but *Pr. juliflora*. and *B. elegans *gave 100% mouse survival.

**Table 4:** *In vivo *activities of plant extracts against *P. berghei*

Plant species	Extract/drug	Dose (mg/kg/day)	Suppression of parasitaemia at day 4 (SP ± SD*)	Survival (%) on day 10	P value (P < 0.05)
***S. surattense***	Dichloromethane	100	58.11 ± 4.3	80	0.035

***Pr. juliflora***	Dichloromethane	100	44.13 ± 1.79	100	0.008
	
	Water-soluble extract	50	2.59 ± 1.54	100	0.598

***B. elegans***	Dichloromethane	300	66.18 ± 2.11	100	0.007

	PBS	-	0	100	

	DMSO	18%	0	100	

	CQ	25	100	100	

* SD = Standard deviation

## Discussion

In this investigation, anti-plasmodial activity of 10 Iranian traditional selected plants, representing five families was studied. The anti-plasmodial activities of the extracts were qualified as "active" when IC_50 _is ≤ 50 μg/ml. Extracts having activity beyond this range were considered inactive. The selectivity index (SI) is defined as the ratio of the cytotoxicity on the brine shrimp to the anti-plasmodial activity. Those that showed high SI (>10) should offer the potential for safer therapy.

Among 10 plants, three species, *Pr. juliflora*. *B. elegans *and *S. surattense *showed promising anti-plasmodial activity with IC_50 _values of ≤ 50 μg/ml and SI ranging between 18.19 and 349.71. From these three species, two (*Pr. juliflora *and *B. elegans*) were found to be highly active with IC_50 _values of < 20 μg/ml. The crude extract of these three active herbs was further fractionated with a mixture of water and dichloromethane. The dichloromethane fraction of three active plants showed anti-plasmodial activity, but only the aqueous phase of *Pr. juliflora *showed activity against two strains of *P. falciparum*. Fortunately, these three active plants had low toxicity indicating the possibility of using them as lead structures in further studies.

**Figure 2:**
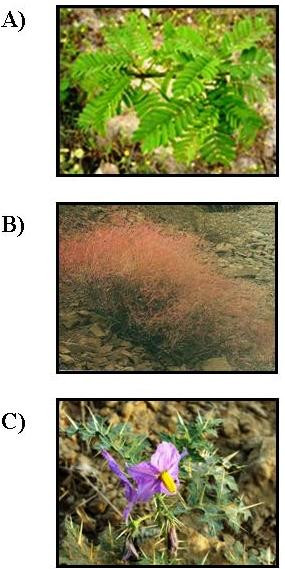
**Image of A: *Prosopis juliflora *called "Somr" in Khouzestan province**. B: *Boerhavia elegans*, called "Sourkho" in Sistan and Baluchistan province; C: *Solanum surattense *called "Tajrizi Baluchi" in Sistan and Baluchistan province, Iran. All these plants showed *in vitro *and *in vivo *anti-plasmodial activity.

*Prosopis juliflora *(IC_50 _= 14.78 μg/ml on K1 strain, SI = 110.73 and IC_50 _= 4.68 μg/ml on CY27 strain, SI = 349.71) is a tree native to Mexico, South America and Caribbean that later become established as a weed in Asia, Australia and elsewhere (Figure 2A). *Pr. juliflora *has been used in traditional medicine for fever, inflammation, scurvy, as a disinfectant, healing of wounds and skin problems [26]. The anti-malarial activity of this plant against CQ-susceptible strain of *P. falciparum *(3D7) was previously reported [27] and the present result correlates well with that and our study confirmed this observation with higher activity than previous study against both CQ-resistant (K1) and -sensitive (CY27) strains. Furthermore, treatment with the dichloromethane extract of *Pr. juliflora *significantly inhibited parasitaemia of *P. berghei *infection in BALB/c mice compared to no treatment, implying direct parasiticidal activity. The water-soluble extract of *Pr. juliflora *was active in *in vitro *against both sensitive and resistance *P. falciparum *strains, but had no *in vivo *activity against *P. berghei*. The results for *in vivo *anti-malarial activity do not necessarily correlate with those for *in vitro *anti-malarial activity as reported before [28] and this may be due to poor bioavailability of the active compounds *in vivo*.

*Boerhavia elegans *is an erect herb up to 1 m high, with a stout rootstock. The stems fleshy become woody towards the base, which is green, often flushed with red, glabrescent to pubescent, branching mainly from the base with the nodes swollen. Its leaves broadly ovate to lanceolate, green above, grayish-white beneath, sometimes tinged purple (Figure 2B). It showed promising anti-plasmodial activity with an IC_50 _= 15.33 μg/ml on K1 strain (SI = 18.19) and IC_50 _= 11.97 μg/ml on CY27 strain (SI = 23.3). 	The dichloromethane extract of *B. elegans *had the highest suppressive activity on *P. berghei *(66.18%) at a concentration of 300 mg/kg/per day. The result has shown promising suppressive and curative anti-malarial activities in *P. berghei *infected mice.

*Solanum surattense *is a suffrutescent perennial under-shrub plant, which grows in dry situations throughout India. The zigzag branches spread close to the ground covered with strong, broad and sharp yellowish white prickles. Its leaves armed with sharp prickles, blue flowers, fruit berries, yellow or white (Figure 2C). This species also showed anti-plasmodial activity with an IC_50 _= 50 μg/ml on K1 strain (SI = 61.32) and IC_50 _= 40.88 μg/ml on CY27 strain (SI = 48.86) and *in vivo *anti-plasmodial activities with 58.11% suppression.

*B. elegans *and *S. surattense *are growing in the south-eastern of Iran, where malaria is endemic. In Sistan and Baluchistan province, people use the extract of *S. surattense *against fever and infections. Also, this plant and *B. elegans *are used for inflammation in folk medicine [29]. No biological properties have been reported in the literature for these species, and the anti-plasmodial activity of these two plants is reported for the first time in the present study. It is interesting to note that the majority of the aqueous extracts did not show any activity. However, the dichloromethane fraction of three extracts showed better anti-plasmodial activity than the total extracts.

In this study, a number of selected plants did not display *in vitro *anti-plasmodial activity. Among them, *Acacia farnesiana *was collected from Khouzestan province in Iran and showed no anti-plasmodial activity. However, an ethanolic extract of this plant from Colombia had shown anti-plasmodial activity against *P. falciparum *[30]. A possible explanation could be that factors such as chemotypes, environmental parameters, harvesting and storage conditions that could collectively influence the plant metabolites prior to and following harvestings, which in turn, would be reflected in the bioactivity.

## **Conclusion**

Overall, increasing the global spread of multi-drug resistant malaria parasite showed that there is a need for new chemotherapeutic agents to combat malaria. In this study, aiming to search for new anti-malarial drugs, we found, for the first time, *in vitro *and *in vivo *anti-plasmodial activities of the plants *B. elegans *and *S. surattense*. The plant *Pr. juliflora *has already been described in the literature for anti-malarial activity against CQ-sensitive *P. falciparum *strain and the present study also confirmed these observations against CQ-resistant *P. falciparum *strain as well as on *P. berghei*. The present finding is only preliminary, the next step will be to isolate and identify the active compound of both *B. elegans *and *S. surattense*.

## Competing interests

The authors declare that they have no competing interests.

## Authors' contributions

AR (PhD student) contributed in the laboratory work, analysis of the data and drafted the paper. SZ designed the work and supervised the *in vitro *and *in vivo *studies, analysed the data and wrote the manuscript. SS contributed in making plant target list, collection and identification of plants, extraction and toxicity design. NK contributed in plant data gathering. NDD helped with the preliminary analysis of the data and also critical reading of the manuscript. All authors contributed to the critical review of the manuscript and agree to submission.
